# Patient Complications after Interscalene Block: A Retrospective Comparison of Liposomal Bupivacaine to Nonliposomal Bupivacaine

**DOI:** 10.1155/2020/6704303

**Published:** 2020-03-27

**Authors:** Jacob L. Hutchins, Jason Habeck, Zac Novaczyk, Richard Campbell, Christopher Creedon, Ellen Spartz, Michael Richter, Jeremy Wolter, Gaurav Suryawanshi, Alexander Kaizer, Aaron A. Berg

**Affiliations:** ^1^Department of Anesthesiology, University of Minnesota, Minneapolis, MN, USA; ^2^Department of Biostatistics and Informatics, Colorado School of Public Health, University of Colorado-Anschutz Medical Campus, Aurora, CO, USA

## Abstract

**Background:**

The purpose of this study was to investigate if the addition of liposome bupivacaine (LB) to an interscalene block (ISB) had an effect on the number of patients with surgical- or block-related complications.

**Methods:**

This was a single-center retrospective chart view performed by identifying patients who received an ISB from January 1, 2014, through April 26, 2018, at the University of Minnesota. 1,518 patients were identified who received an ISB (LB = 784, nonliposomal bupivacaine = 734). Patients were divided into two groups those who did receive liposome bupivacaine in their ISB and those who did not receive liposome bupivacaine in their ISB. Medical records were individually reviewed for surgical procedure, block medications, complications related to the block or surgical procedure, phone calls to the healthcare system for issues related to opioids or pain within 3 and within 30 days, readmissions within 30 days, and emergency room visits for complications within 3 and 30 days.

**Results:**

There was no significant difference in the number of patients with surgical or anesthetic complications. Only phone calls for pain within 3 days were significantly different. The LB group had 3.2% of patients call compared to 5.6% in the nonliposomal bupivacaine group (aOR = 1.71 (95% CI: 1.04–2.87), *p*=0.036). We found no significant difference in any of the other secondary outcomes.

**Conclusions:**

The use of LB in an ISB demonstrated no significant difference compared to nonliposomal bupivacaine in numbers of complications, emergency room visits, and readmissions.

## 1. Background

Patients commonly experience moderate-to-severe postoperative pain after undergoing shoulder surgery [1]. Traditionally, postoperative pain had been managed with opioids because of their effectiveness in controlling moderate-to-severe pain; however, adverse effects such as nausea, vomiting, and constipation are common [2]. Opioids can lead to persistent use and dependence, and they are a direct cause of respiratory depression, which can lead to overdose and death [3]. Patients are becoming increasingly concerned about their postoperative pain management and the use of opioids [4]. As a result, the use of regional anesthesia techniques such as interscalene blocks (ISBs) is increasing.

Analgesia for shoulder procedures can be safely and effectively achieved through the use of regional anesthesia [5, 6]. ISBs are commonly performed in shoulder surgery, which aim to block the proximal trunk of the brachial plexus. ISBs have been shown to be a safe and effective method for analgesia during shoulder procedures [5–8]. When compared to general anesthesia alone, ISBs have been shown to decrease recovery room stay, nonsurgical intraoperative time, and intraoperative narcotics [9, 10]. ISBs have also been shown to result in improved early postoperative pain control, decreased unplanned hospital admissions secondary to severe pain, decreased nausea/vomiting, and decreased duration of hospital stay [8, 10, 11]. Complications of ISBs include brachial plexus injury, nerve damage, local anesthetic systemic toxicity, Horner's syndrome, and phrenic nerve paralysis [12–15].

More recently, liposomal bupivacaine (LB) (EXPAREL®; Pacira Pharmaceuticals, Inc; Parsippany, NJ, USA) has been developed to increase the duration of analgesia for both infiltration and regional anesthesia. Compared to standard bupivacaine, LB used in ISBs for rotator cuff repair (RCR) or total shoulder arthroplasty (TSA) was associated with no difference in adverse events, decreased pain, and higher patient satisfaction in the first week following surgery [16]. Sun et al. performed a systematic review and found that LB was associated with a lower complication rate when compared to other local anesthetics used in an ISB [17].

The purpose of this study was to determine if there were any differences in the number of patients with surgical- or block-related complications with and without the addition of LB. Additionally, we wanted to understand if the use of liposomal bupivacaine in an ISB impacted the number of readmissions, phone calls, or emergency department (ED) visits for patients undergoing shoulder surgery. We hypothesized that there would be no difference in postoperative complications, pain phone calls, and ED visits between blocks performed with added LB versus those without.

## 2. Methods

This was an Institutional Review Board (IRB) exempt study (IRB ID STUDY00003305) as it involved research involving the collection or study of existing data, documents, and records in which the information recorded by the research team was such that the subjects could not be identified. The requirement for written informed consent was waived by the IRB. This manuscript adheres to the applicable STROBE guidelines. This was a single-center retrospective chart review of patients who received interscalene brachial plexus blocks at the University of Minnesota from January 1, 2014, to April 28, 2018. Inclusion criteria included American Society of Anesthesiologists (ASA) I-III patients 18 years of age or older who received a preoperatively placed interscalene single shot block for postoperative pain. Patients were excluded if they declined access to their medical charts for research purposes when asked on admission, received an interscalene catheter, or had a confirmed diagnosis of chronic pain in their medical record. Electronic medical charts were individually reviewed for surgical procedure, block medications used, complications related to the block or surgical procedure, phone calls to the healthcare system for issues related to opioids or pain within 3 days and within 30 days, readmissions within 30 days, and emergency room visits for pain, opioids, or block-related complications within 3 days and within 30 days. All notes in the patient chart from the day of surgery until 30 days after surgery were reviewed. Opioid-related issues included calls or visits due to side effects from opioids such as constipation, nausea/vomiting, urinary retention, intolerance of the medication, opioid overdose, and need for more opioid medication were recorded. Block-related complications included but were not limited to shortness of breath, prolonged numbness in the affected extremity, or Horner's syndrome. Patients presenting with pain to the emergency room or reporting pain control as an issue during a phone call were recorded as uncontrolled pain. Demographic data such as age, sex, weight, and ASA status was also documented. Patients were then divided into liposomal bupivacaine and nonliposomal bupivacaine groups for statistical analysis. Those patients in whom the medications used for the block were recorded but volumes of medications used were not recorded were included in the study. Those patients in whom there was no mention of what medication was used for their ISB were excluded in the final analysis. The primary outcome of this study was the number of patients with postoperative block and surgical complications seen in each group. Secondary outcomes were rates of readmission, rates of emergency room visit related and unrelated to the ISB, pain, and opioids, and phone calls to the healthcare system related and unrelated to the ISB, pain, and opioids.

### 2.1. Statistical Analysis

Continuous measures were summarized as mean (standard deviation), and categorical measures were summarized as count (%) with a two-sample *t*-test or chi-squared test to detect any significant differences between the liposomal bupivacaine group and nonliposomal bupivacaine groups, respectively. As all outcomes were binary, Firth's penalized logistic regression was used to compare groups to take into account the small sample sizes of events and potential bias of parameter estimates, adjusting for any significant baseline measures (*p* < 0.05). The *p* values are not adjusted for multiple comparisons and should be interpreted cautiously. All analyses completed with R version 3.5.1 [18], and Firth's logistic regression used the *logistf* package [19].

## 3. Results

1,869 ISBs were identified to have occurred from January 1, 2014, through April 26, 2018. After applying the inclusion and exclusion criteria, a total of 1,518 patients were included in the analysis. 246 patients were excluded due to not allowing their charts for research, and 105 were excluded due to being under age 18, ASA IV, having a continuous catheter, incomplete data regarding what medication was used for the block, and having a diagnosis of chronic pain in their medical record. 784 patients received liposomal bupivacaine, and 734 patients received a nonliposomal bupivacaine block. The nonliposomal bupivacaine group had significantly more male patients than the liposomal bupivacaine group (*p* < 0.001). The liposomal bupivacaine group had significantly more ASA 3 and less ASA 1 compared to the nonliposomal bupivacaine group (*p*=0.010) All other baseline characteristics were similar (Table 1).

There was a wide variation in the combination of local anesthetics used in the two groups. In the liposomal bupivacaine group, the combination of liposomal bupivacaine along with 0.25% bupivacaine + epinephrine (*N* = 229) and liposomal bupivacaine + 0.5% bupivacaine + epinephrine (*N* = 225) were the most common. The median volume of total local anesthetic used was 20 mL for the liposomal bupivacaine group. The median volume of liposomal bupivacaine used was 10 mL, and median volume of nonliposomal bupivacaine used was 10 mL (Table 2).

The patients in the nonliposomal bupivacaine group received either bupivacaine or ropivacaine as the primary agent of their block. The most common combination in this group was 0.5% ropivacaine with clonidine and epinephrine (*n* = 387). The median volume of the interscalene was 25 mL in this group (Table 3).

Table 4 shows the occurrence of outcomes and the odds ratios estimated from Firth logistic regression results for comparisons between the two groups, adjusting for sex and ASA status. There were 25 patients in the liposomal bupivacaine group with complications and 22 in the nonliposomal bupivacaine group (aOR = 0.95 (95% CI: 0.53–1.70), *p*=0.873). There were 5 patients with cardiopulmonary complications observed in the nonliposomal bupivacaine group (postoperative atrial fibrillation, intraoperative hemodynamic instability, bradycardia, aspiration, ST-segment elevation, and pulmonary embolus). There were seven patients with postoperative numbness, three postoperative infections, two with uncontrolled nausea/vomiting, one postoperative bleed, one with axillary nerve paresthesias, one patient with postoperative shortness of breath, and one with postoperative delirium.

In the liposomal bupivacaine group, there were eight patients with prolonged postoperative numbness, seven patients with infection, two patients with uncontrolled nausea/vomiting, two patients with shortness of breath, two with retear needing repair, one postoperative bleed, one with a pulmonary embolus, one with acute lower extremity weakness, and one with bilateral hand swelling and weakness.

Of the secondary outcomes evaluated, only calls for pain within 3 days were significant. The liposomal bupivacaine group had 3.2% of patients call compared to 5.6% in the nonliposomal bupivacaine group (aOR = 1.71 (95% CI: 1.04–2.87), *p*=0.036).

Tables 5 and 6 describe the reasons for emergency room visits for opioid or pain-related issues within 3 and 30 days, while Tables 7 and 8 report reason for phone calls over the same time frame. Among both groups, arthroscopic rotator cuff repair (LB 36.0% vs. non-LB 38.6%), total shoulder arthroplasty (LB 15.6% vs. non-LB 14.6%), and reverse total shoulder arthroplasty (LB 11.4% vs. non-LB 9.3%) were the most common procedures performed (Table 9).

Additionally, we explored three sets of subgroup analyses for the results of Table 4 for the block types of bupivacaine 0.25%, bupivacaine 0.25% with epinephrine 1 : 200000, and bupivacaine 0.50% with epinephrine 1 : 200000. Given the focus on each specific combination, the total volume administered was also included in Firth's logistic regression models in addition to sex and ASA status. Given the smaller sample sizes within each subgroup, we generally observed insignificant results for comparing liposomal bupivacaine and the nonliposomal bupivacaine group, with the exception of calls for pain within 30 days for the bupivacaine 0.25% with epinephrine 1 : 200000 subgroup, where the liposomal bupivacaine group had 23.8% of patients call compared to 9.8% in the nonliposomal bupivacaine group (aOR = 0.43 (95% CI: 0.16–0.99), *p*=0.047).

## 4. Discussion

Compared to nonliposomal bupivacaine single shot ISBs, there was no difference in the number of patients with block- or surgical-related complications. Of the block-related complications in both groups, all patients with postoperative numbness resolved within 30 days, and the SOB resolved by postoperative day 2. Our study results are comparable to data from several large database studies and meta-analysis on nonliposomal bupivacaine interscalene blocks. The meta-analysis by Moore et al. looked at 6243 interscalene blocks and found a major complication rate of 0.35% and a minor complication rate of 11.32% [20]. Shin et al. performed a large database study on 27,072 shoulder arthroscopies and found a surgical complication rate of 7.9% and block-related complication rate of 0.59% [21]. Koh et al. evaluated 11,450 shoulder arthroplasty patients finding a complication rate of 7.6% [22]. While the rate of complications seen in our study is similar to the large database meta-analyses [20–22], all are still lower than described by Vandepitte et al. who compared liposomal bupivacaine added to bupivacaine vs. bupivacaine interscalene blocks [16]. They found that 17 out of 26 patients in the liposomal bupivacaine group reported adverse events compared to 10 out of 24 in the bupivacaine group. All adverse events occurred within the first 24 hours and reported no further adverse events. There was 0% in the liposomal bupivacaine group with shortness of breath compared to 16% in the bupivacaine group. While these rates of adverse events are much higher than the number of patients with complications that we saw in our study and in the previous large database studies, this is likely due to the retrospective nature of our study and differences in documentation of adverse events.

Postoperative pain-related emergency room visits are important to surgical teams in patient satisfaction and cost to the hospital. The most common reason for visiting the emergency room in this study was for uncontrolled pain, with other reasons including urinary retention, constipation, and numbness. In the acute setting (within 3 days of surgery), the bupivacaine liposomal group had 1.0% of patients return to the ED, while the nonliposomal bupivacaine group had 1.5%. There was also no difference in patients visiting the emergency room within 30 days, with visits from 1.4% of liposomal bupivacaine patients and 1.5% of non-liposomal bupivacaine patients. Navarro et al. found that there was 90/1306 or 6.9% unplanned emergency room and urgent care visits within 7 days after outpatient rotator cuff repair. Of those 90, 34 (or 2.6%) were related to pain, which is similar to our findings [23].

There was also a similarity in hospital readmissions between the liposomal bupivacaine and nonliposomal bupivacaine group, with 2.4% and 2.9% readmissions, respectively. Additionally, there were 19 readmissions in the liposomal bupivacaine group, which indicates that liposomal bupivacaine is not masking serious complications that require hospital attention. These readmission rates are in line with previously reported readmissions rates following shoulder surgery. Both Shin et al. and Kosinski et al. found a 1% 30-day readmission rate for patients undergoing outpatient rotator cuff repairs, whereas Koh et al. and Cvetanovich et al. found a 30-day readmission rate of 2.3% and 2.56%, respectively, in patients undergoing shoulder arthroplasty [21, 23–25].

Postoperative phone calls from surgical patients may indicate inadequate pain control, which can ultimately require increased time demand from surgeons. Our study demonstrates that there were significantly fewer patient calls in the liposomal bupivacaine group compared to the nonliposomal bupivacaine group within three days after surgery. The most common reason for phone calls within the liposomal bupivacaine group was for pain medication refills, indicating a continued need for pain control. The number of calls for pain medication refills between the two groups was similar (13 in the liposomal bupivacaine group and 14 in the nonliposomal bupivacaine group). In contrast, the most common reason for phone calls in the nonliposomal bupivacaine group was uncontrolled pain. Phone calls reporting symptoms such as constipation or numbness were scarce and similar in both groups. The similarity in the number of phone calls within 30 days of surgery is expected since the effect of both blocks has worn off.

It is important to note that the baseline characteristics of the two groups were not similar. The nonliposome group had significantly more males than the liposomal bupivacaine group, and the liposomal bupivacaine group had significantly more ASA 3 and significantly less ASA 1 than the nonliposomal bupivacaine group. These may impact the results as having more males may increase the risk of nerve injury as well as having increased ASA level [26, 27].

Limitations of this retrospective study include the wide variety of medication combinations used, including medication type and volume, in both the liposomal bupivacaine and nonliposomal bupivacaine groups. While the wide variety of procedures in this study was beneficial in indicating medication safety, they could impact statistical analysis as variables. Postoperative complications were followed for 30 days and thus may not provide a complete assessment between rates of complications between the two study groups. Additionally, any out-of-network phone calls, ER visits, or admissions were not captured by this study.

## 5. Conclusions

The use of liposomal bupivacaine for interscalene blocks demonstrated no significant difference in the number of patients with surgical or block complications when compared to nonliposomal bupivacaine. While there was a significant difference in the number of patients that made a phone call about their pain by postoperative day 3, there was no difference in any of the other secondary outcomes. Thus, further prospective studies are warranted to demonstrate the safety and potential efficacy of liposomal bupivacaine in ISB for shoulder operations.

## Figures and Tables

**Table 1:** Baseline characteristics between the two groups.

Covariate	LB (*N* = 784)	Non-LB (*N* = 734)	*p* value
Age (years)	56.4 (15.4)	55.0 (15.8)	0.095
Weight (kg)	87.8 (21.3)	88.5 (21.1)^1^	0.518
Male	392 (50%)	430 (58.6%)	<0.001
ASA 1	118 (15.1%)	149 (20.3%)	0.010
ASA 2	412 (52.6%)	384 (52.3%)	
ASA 3	254 (32.4%)	201 (27.4%)	

^1^One non-LB weight observation missing. LB: liposomal bupivacaine. Non-LB: nonliposomal bupivacaine. Continuous covariates presented mean (standard deviation) with a *p* value from two-sample *t*-test, categorical covariates presented as count (%) with *p* value from chi-squared test.

**Table 2:** Varying medication combinations in the liposomal bupivacaine group.

Drug combination	Volume (mL) range	Volume (mL) median	Number missing
LB + 0.25% bupivacaine (*N* = 130)			

LB	5–20	10	2
0.25% bupivacaine	2–20	10	1

LB + 0.25% bupivacaine plus epinephrine (*N* = 229)			

LB	5–20	10	1
0.25% bupivacaine plus epinephrine	2–25	10	0

LB + 0.5% bupivacaine (*N* = 118)			

LB	5–20	10	1
0.5% bupivacaine	3–20	10	0

LB + 0.5% bupivacaine plus epinephrine (*N* = 225)			

LB	7.5–20	10	0
0.5% bupivacaine plus epinephrine	3–20	10	0

LB only (*N* = 79)			

LB	10–20	15	0

LB + 0.5% ropivacaine plus clonidine plus epi (*N* = 3)			

LB	10–15	10	0
0.5% ropivacaine plus clonidine plus epi	10–15	10	0

LB: liposomal bupivacaine; *N*: the number of patients included in that group.

**Table 3:** Medication combinations in the nonliposomal bupivacaine group.

Drug combination	Volume (mL) range	Volume (mL) median	Number missing
0.25% bupivacaine (*N* = 27)	5–40	25	0
0.25% bupivacaine plus epinephrine (*N* = 64)	10–40	27.5	0
0.3–0.33% bupivacaine (*N* = 10)	30–40	35	0
0.375% bupivacaine (*N* = 1)	30	30	0
0.5% bupivacaine (*N* = 43)	10–40	20	0
0.5% bupivacaine plus epinephrine (*N* = 115)	5–40	20	1
0.5% ropivacaine (*N* = 52)	12–40	25	0
0.5% ropivacaine plus clonidine plus epinephrine (*N* = 387)	15–40	25	33
0.5% ropivacaine plus epinephrine (*N* = 23)	20–35	25	0
0.5% ropivacaine plus 8 mg dexamethasone (*N* = 3)	30	30	0
0.25% bupivacaine plus 8 mg dexamethasone (*N* = 2)	19–20	19.5	0
Mixture of bupivacaine and ropivacaine (*N* = 7)	20–50	25	0

*N*: number of patients included in that group.

**Table 4:** Firth's logistic regression for primary and secondary outcomes with number experiencing the outcome with % within group and *p* value for comparison of two groups.

Outcome	LB *N* (%) with outcome	Non-LB *N* (%) with outcome	aOR (95% CI) (non LB = 1)	*p* value
Surgical or anesthetic complication	25 (3.2%)	22 (3.0%)	0.95 (0.53, 1.70)	0.873
Readmission	19 (2.4%)	21 (2.9%)	1.31 (0.70, 2.46)	0.403
ER pain within 3 days	8 (1.0%)	11 (1.5%)	1.38 (0.56, 3.49)	0.482
ER within 30 days	42 (5.4%)	55 (7.5%)	1.47 (0.97, 2.24)	0.068
ER pain within 30 days	11 (1.4%)	11 (1.5%)	1.05 (0.45, 2.43)	0.907
Call pain within 3 days	25 (3.2%)	41 (5.6%)	1.71 (1.04, 2.87)	0.036
Call within 30 days	257 (32.8%)	240 (32.7%)	1.01 (0.81, 1.25)	0.919
Call pain within 30 days	155 (19.8%)	143 (19.5%)	1.00 (0.77, 1.29)	0.983

LB: liposomal bupivacaine, non-LB: nonliposomal bupivacaine, ER: emergency room, and aOR: adjusted odds ratio.

**Table 5:** Emergency room visit for opiate or pain-related reason within three days of surgery.

Reason for visit	LB group	Non-LB group
Uncontrolled pain	7	5
Urinary retention	1	5
Constipation	0	1
Total	8	11

LB: liposomal bupivacaine.

**Table 6:** Emergency room visit for opiate or pain-related reason within 30 days of surgery.

Reason for visit	LB group	Non-LB group
Uncontrolled pain	9	5
Urinary retention	1	5
Constipation	0	1
Numbness	1	0
Total	11	11

LB: liposomal bupivacaine.

**Table 7:** Phone call for opiate or pain-related reason within three days of surgery.

Reason for call	LB group	Non-LB group
Shortness of breath	1	1
Numbness	5	3
Uncontrolled pain	5	19
Pain medications	13	14
Urinary retention	1	2
Pruritis	0	1
Constipation	0	1
Total	25	41

LB: liposomal bupivacaine.

**Table 8:** Phone call for opiate or pain-related reason within 30 days of surgery.

Reason for call	LB group	Non-LB group
Uncontrolled pain	16	24
Pain medications	121	102
Numbness	8	7
Constipation	1	0
Pruritis	0	1
Shortness of breath	0	1
More than 1 of listed reason	9	8
Total	155	143

LB: liposomal bupivacaine.

**Table 9:** Procedure summary table; values presented are *N* (%).

Covariate	Overall	LB	Non-LB
	(*N* = 1518)	(*N* = 784)	(*N* = 734)
Arthrodesis	2 (0.1%)	2 (0.3%)	0 (0.0%)
Arthroscopic bankart/labral	74 (4.9%)	27 (3.4%)	47 (6.4%)
Arthrosocopy arthrodesis	1 (0.1%)	0 (0.0%)	1 (0.1%)
Arthrosocopy biceps	68 (4.5%)	27 (3.4%)	41 (5.6%)
Arthrosocopy biopsy	12 (0.8%)	5 (0.6%)	7 (1.0%)
Capsular repair	17 (1.1%)	9 (1.1%)	8 (1.1%)
Clavicle fracture	22 (1.4%)	4 (0.5%)	18 (2.5%)
Distal clavicle	1 (0.1%)	0 (0.0%)	1 (0.1%)
Elbow fracture	3 (0.2%)	1 (0.1%)	2 (0.3%)
Hardware removal	59 (3.9%)	41 (5.2%)	18 (2.5%)
Humerus fracture	46 (3.0%)	28 (3.6%)	18 (2.5%)
I and D	14 (0.9%)	6 (0.8%)	8 (1.1%)
Manipulation/reduction	6 (0.4%)	0 (0.0%)	6 (0.8%)
Open biceps	2 (0.1%)	1 (0.1%)	1 (0.1%)
Open instability surgery	23 (1.5%)	19 (2.4%)	4 (0.5%)
Open RCR	1 (0.1%)	0 (0.0%)	1 (0.1%)
RCR	565 (37.2%)	282 (36.0%)	283 (38.6%)
Resurfacing	2 (0.1%)	1 (0.1%)	1 (0.1%)
Reverse TSA	157 (10.3%)	89 (11.4%)	68 (9.3%)
Reverse TSA revision	49 (3.2%)	36 (4.6%)	13 (1.8%)
Simple arthroscopy	139 (9.2%)	68 (8.7%)	71 (9.7%)
Triceps	1 (0.1%)	1 (0.1%)	0 (0.0%)
TSA	237 (15.6%)	130 (16.6%)	107 (14.6%)
TSA revision	14 (0.9%)	5 (0.6%)	9 (1.2%)

LB: liposomal bupivacaine.

## Data Availability

The data sets generated and analysed during the current study are not publicly available due to the IRB but are available from the corresponding author on reasonable request.

## Abbreviations

ISB: Interscalene block
LB: Liposome bupivacaine
RCR: Rotator cuff repair
TSA: Total shoulder arthroplasty
IRB: Institutional Review Board
ED: Emergency department
ASA: American Society of Anesthesiologists.
